# A Nonhost Peptidase Inhibitor of ~14 kDa from *Butea monosperma* (Lam.) Taub. Seeds Affects Negatively the Growth and Developmental Physiology of *Helicoverpa armigera*

**DOI:** 10.1155/2014/361821

**Published:** 2014-04-17

**Authors:** Prabhash K. Pandey, Dushyant Singh, Sangram Singh, M. Y. Khan, Farrukh Jamal

**Affiliations:** ^1^Department of Biochemistry [DST-FIST & UGC-SAP Supported], Dr. Ram Manohar Lohia Avadh University, Faizabad, Uttar Pradesh 224001, India; ^2^Department of Biotechnology, Babasaheb Bhimrao Ambedkar University, Lucknow, Uttar Pradesh 226025, India

## Abstract

*Helicoverpa armigera* is one of the major devastating pests of crop plants. In this context a serine peptidase inhibitor purified from the seeds of *Butea monosperma* was evaluated for its effect on developmental physiology of *H. armigera* larvae. *B. monosperma* peptidase inhibitor on 12% denaturing polyacrylamide gel electrophoresis exhibited a single protein band of ~14 kDa with or without reduction. *In vitro* studies towards total gut proteolytic enzymes of *H. armigera* and bovine trypsin indicated measurable inhibitory activity. *B. monosperma* peptidase inhibitor dose for 50% mortality and weight reduction by 50% were 0.5% w/w and 0.10% w/w, respectively. The IC_50_ of *B. monosperma* peptidase inhibitor against total *H. armigera* gut proteinases activity was 2.0 µg/mL. The larval feeding assays suggested *B. monosperma* peptidase inhibitor to be toxic as reflected by its retarded growth and development, consequently affecting fertility and fecundity of pest and prolonging the larval-pupal duration of the insect life cycle of *H. armigera*. Supplementing *B. monosperma* peptidase inhibitor in artificial diet at 0.1% w/w, both the efficiencies of conversion of ingested as well as digested food were downregulated, whereas approximate digestibility and metabolic cost were enhanced. The efficacy of *Butea monosperma* peptidase inhibitor against progressive growth and development of *H. armigera* suggest its usefulness in insect pest management of food crops.

## 1. Introduction

Every creature plays a vital role in the sustenance of any natural ecosystem. Synthetic chemicals such as organochlorines, organophosphates, and pyrethroids used as weedicides and pesticides in farming are toxic and nonselective in their action. Therefore, deployment of chemical approaches not only eradicates selected targeted pests but also adversely affects other organisms causing serious damage to the ecosystem. Moreover, major fallout of this approach of pest management is acclimatization of pests against such chemicals leading to a gradual increase in the lethal dosage [1]. The cotton bollworm,* H. armigera* Hübner (Lepidoptera: Noctuidae), is a serious rapacious feeder pest, adaptable on diverse hosts of economically significant cropping systems due to its high fecundity and worldwide prevalence [2]. Transgenic approaches involving the expression of ‘‘*cry* toxin genes” (encoding family of crystalline toxins) from* Bacillus thuringiensis *in plants have shown considerable acceptability in controlling* H. armigera*.

The rampant use of chemically synthesized insecticides currently used to control insect damage in agriculture incurs an enormous cost worldwide and with a strong adverse environmental impact. Therefore, genetic engineering of crop plants leading insect tolerance is of paramount interest to agricultural biotechnologists. Ectopic expressions of genes coding for proteinase inhibitors, which are part of the natural defence system developed by plants against insect attack, represent an effective approach. Successful protection against insect pests has been observed in different transgenic plants which contain genes coding for proteinase inhibitors [3, 4]. Moreover, resistance development against few available genes/chemicals imposes serious limitations on its wider usage. Thus, it is essential to investigate and select appropriate alternatives such as proteinase inhibitors (PIs) which upon expression in transgenic plants will be successful in conferring protection against the target pest in a sustainable manner. Even though PIs are promising molecules, the selection pressure exerted by the rigidity of PIs compels the pests to adapt to such stressful situations by expanding the repertoire of functionally similar proteinases exhibiting insensitivity to these PIs [5] or with an altered capability of digesting different substrates efficiently [6]. This flexible adaptation is responsible for the pest's remarkable success in which their biochemical weaponry is diversified to combat the host defence.

Serine PIs have gained importance due to their ubiquitous distribution in the plant kingdom [7] and the dependence of lepidopteran pests including* Spodoptera litura* and* H. armigera* on serine proteinases for metabolism of food proteins [8]. PIs are widely distributed in plant seeds, where they act as antinutritional agents, especially in insects where they inhibit midgut proteinases [7, 8] by reacting with cognate enzymes and binding in a canonical fashion to catalytic sites [9]. The presence of an exposed loop in all canonical inhibitors simulates a rapid binding and a slow dissociation mechanism [10].

Host-pest interactions leading to coevolution of host resistance and pest adaptation have resulted in the development of novel and unique strategies for both plants and pests to overcome each other's defence capabilities [11].* B. monosperma *(flame of forest, common name: Dhak, Palash) is a medium-sized deciduous tree belonging to family Leguminosae (Papilionaceae). It grows throughout the Indian subcontinent, especially in Indo-Gangetic plains. The plant is useful in many ways and the seeds are used in Ayurvedic, Homeopathic, and Unani medicines for treating a number of human maladies. Keeping the significance of the coevolving host-pest interaction, we have studied the effectiveness of a nonhost serine peptidase inhibitor from* B. monosperma* against the generalist feeder* H. armigera* of several economically important crops.

Larval stage is very crucial for accumulating nutrients and energy, which is used for pupal and adult development, fertility, and fecundity. Early instars usually feed on low nutrient leaves and progressively consume nutrient rich reproductive structures of plants. The larvae survive even in highly adverse conditions due to their adaptive capabilities including polyphagy, high fecundity, mobility, and facultative diapauses [12]. The gut proteinase expression system of* H. armigera* larvae synthesizes new and higher amounts of proteinases during starvation and added stress could possibly account for arrested growth and mortality [13]. The goal of the present work was to purify and evaluate the bioinsecticidal and growth inhibitory activity of a peptidase inhibitor from* B. monosperma* seeds (BmPI) on* H. armigera*. Further, the effect of inhibitory activity of BmPI on growth and development of* H. armigera* has been demonstrated using three different doses of inhibitor in artificial diet.

## 2. Materials and Methods

### 2.1. Materials

Mature dry seeds of* B. monosperma* were collected from Dr. R.M.L. Avadh University Campus, Faizabad (Uttar Pradesh, India). The plant was authenticated by plant taxonomist Dr. Tariq Husain, National Botanical Research Institute, Lucknow. The voucher specimen (specimen accession number 98180-2011) was deposited at the NBRI herbarium. N-tosyl-L-phenylalanine chloromethyl ketone (TPCK) treated bovine trypsin, synthetic substrate N-*α*-benzoyl-DL-arginine-p-nitroanilide (BApNA), standard inhibitor phenyl methyl sulphonyl fluoride (PMSF), acrylamide, and other electrophoretic reagents were purchased from Sigma Chemical Co. (St. Louis, Mo, USA). Sephadex G-75 was purchased from Amersham Biosciences (Uppsala, Sweden). Trypsin-Sepharose CL-4B, azocasein, and protein molecular weight markers were purchased from Sisco Research Limited (Mumbai, India). All other chemicals used were of analytical grade.* H. armigera* were from a laboratory colony which was obtained from Narendra-Dev University of Agriculture and Technology, Kumarganj, Faizabad, India. Insects were housed at 28 ± 2°C, 60% relative humidity with a photoperiod of 14 h light and 10 h dark.

### 2.2. Purification of* B. monosperma* Peptidase Inhibitor (BmPI) and Its Inhibitory Assay against Bovine Trypsin

Finely ground, decorticated* B. monosperma* seed flour was freed of fats and pigments by several washes with chilled acetone and hexane and treated with 0.1 M sodium phosphate buffer (1 : 10 w/v), pH 7.6 with occasional stirring at room temperature for 6 h. The homogenate was centrifuged at 12,000 rpm for 20 min at 4°C and phenolic compounds were removed by treatment with 1% (w/v) polyvinylpyrrolidone. Total soluble protein was incubated at 60°C for 30 min and spun at 12,000 rpm for 20 min at 4°C. The inhibitory activity against trypsin was determined in the supernatant containing heat stable protein. Ammonium sulfate salt was added to the supernatant and precipitate formed at 0–30%, 30–65%, and 65–90% saturation was collected as pellets in all fractions (F_0–30_, F_30–65_, and F_65–90_). The pellet was reconstituted in limiting volume of extraction buffer, dialyzed extensively with the same extraction buffer at 4°C using a membrane with a cut-off range (*M*_*r*_ 12,000; Sigma-grade), and lyophilized. The proteinase inhibitory activity [14] and protein content [15] were estimated for each fraction. The fraction F_30–65_, showing a high level of inhibitory activity against trypsin (59%) as compared to F_0–30_ (26%) and F_65–90_ (37%), was applied on Sephadex G-75 column (100 × 2 cm; Amersham column) and equilibrated with several bed volumes of 0.01 M phosphate buffer, pH 7.6. Fractions of 4.5 mL were collected (BIO-RAD Fraction collector-Model-2110) at an initial flow rate of 0.3 mL/min. The void volume of the column was measured by Blue-dextran. The protein was monitored by using a PC-based (JASCO V-550) spectrophotometer at 280 nm. The active fractions from 42 to 58 were pooled, dialyzed, lyophilized, and applied onto a Trypsin-Sepharose CL-4B column (25 × 1.5 cm) preequilibrated with 0.1 M Tris-HCl buffer (pH 7.6), 5 mM CaCl_2,_ and 0.1 M NaCl. The bound proteins were retrieved at an initial flow rate of 30 mL/h using 100 mM HCl solution. The fractions of antitryptic peak (BmPI) were pooled and lyophilized for further analyses.

125 µL of affinity column purified fraction was added to 15 µg of bovine trypsin in 200 µL of 0.1 M Tris-HCl buffer (pH 7.6) and incubated at 37°C in a water bath for 10 min. Residual trypsin activity was measured by adding 1 mL of 1 mM BApNA in prewarmed (37°C) buffer (0.1 M Tris-HCl, pH 7.6) containing 0.02 M CaCl_2_ and incubated for 10 min. The volume of reaction mixture was kept at 2 mL and terminated by adding 2 mL of 10% trichloroacetic acid and centrifuged. The liberated p-nitroaniline in the clear solution was measured at 410 nm [14]. Controls were run simultaneously during the experiments and assays were done in triplicate. Inhibitor activity was calculated by the amount of purified sample required to inhibit 50% of trypsin activity, which is considered as one unit of trypsin inhibition and expressed as trypsin inhibitor units per mg seed protein.

### 2.3. Polyacrylamide Gel Electrophoresis

Trypsin-Sepharose CL-4B affinity column sample with maximum inhibitory activity was resolved on a discontinuous buffer system of 4% stacking gel and a 12% resolving gel [16]. In the wells 60 µL (60 µg) of column sample and 20 µL (as such) of marker volumes were loaded with bromophenol blue as tracking dye. The gel was stained with coomassie brilliant blue R-250 staining solution (0.025% coomassie blue R-250, 40% methanol, and 7% acetic acid) and subsequently destained with solution I (40% methanol, 7% acetic acid) for 30 min followed by solution II (7% acetic acid, 5% methanol) for 2 h with shaking on rocker platform. Gel was photographed and stored in solution of 10% glycerol and 7% acetic acid. Molecular weight of unknown protein was calculated from the Genei gel-doc fire-reader software.

### 2.4. Midgut Preparation

Actively feeding fourth instar larvae of* H. armigera* were cold immobilized, and killed by decapitation to collect the midguts along with their content. The midguts were placed into an iso-osmotic saline (0.15 M NaCl) and stored frozen (−20°C) until needed. Gut enzyme extract was prepared by homogenizing the midguts in ice-cold 0.1 M glycine-NaOH buffer, pH 10.0 (5 guts/ mL buffer). The homogenate was kept for 2-3 h at 10°C and centrifuged at 12,000 rpm for 15 min at 4°C. The supernatant was stored at −20°C and used as a source of gut proteinases. Using 40 µL of HGPs source (showing an equivalent amount of 15 µg of bovine trypsin activity) the proteinase and % proteinase-inhibitory activities with the purified plant sample were performed.

### 2.5. Enzyme Assay against Proteinase Extract from* H. armigera* Larvae

Trypsin and total proteolytic activities in midgut of* H. armigera* larvae were estimated using azocasein as substrate [17]. To determine the 40–50% inhibition of proteinases of* H. armigera* midgut extract different doses of BmPI were mixed with 40 µL of* H. armigera* gut extract (corresponding to 15 µg of bovine trypsin) and incubated at 37°C for 10 min, before addition of substrate to start the reaction [18]. The results were expressed as IC_50_ or percent inhibition relative to controls without inhibitor. All* in vitro* assays were carried out in triplicates.

### 2.6. Stability of BmPI against HGPs

BmPI was incubated with HGPs at 37°C for 45 min and 3 h to confirm its stability against HGPs. PI activity was measured using BApNA as substrate.

### 2.7. Bioassays of* H. armigera* Larvae Fed on Artificial Diet with BmPI

BmPI role on* H. armigera* development was assessed using the artificial medium system as described by Giri and Kachole (1998) [19]. The affinity purified BmPI was supplemented into artificial diet of starved third instar larvae at concentrations of 0.05, 0.1, and 0.5 (% w/w) and incubated over night at 4°C and diet without PI was used as control. Fresh diet was provided on every alternate day. The observations on larval, pupal developmental parameters including fertility (number of eggs/adult female) and fecundity were recorded and compared with the control larval population.

The feeding experiments were started by releasing 52 neonates individually on each of the test diets rearing plates in four replicates. Cumulative mortality was observed from larval to adult stage. Surviving larvae were transferred to rearing trays containing the respective test diets and reared individually to monitor growth and development. Larval weight was taken on every alternate day. After 13th day which is the end of feeding period of larvae growing on normal diet, all the surviving larvae in the test diets were transferred to normal diet (without inhibitor). The recovery of larvae was monitored by recording their weight at regular interval. Pupal weight and adult emergence were determined on the first day following pupation and upon adult eclosion, respectively.

#### 2.7.1. Antifeedant Activity

The purified BmPI at different concentrations was tested for antifeedant activity against* H. armigera* larvae. Single 3 h prestarved fourth instar larvae of* H. armigera* were introduced into petri dishes containing the respective diets. Four replicates were maintained for each treatment with 13 larvae per replicate (total, *n* = 52). Progressive consumption of diet area by the larvae after 24 h feeding was recorded in control and treated plates. The percent antifeedant index was calculated using the formula of Jannet et al. (2000) [20]. Antifeedant index = [(*C* − *T*)/(*C* + *T*)] × 100 (where *C* and *T* indicate the amount of diet eaten by the larvae on control and treated plates, resp.). The effective dose for a 50% response (ED_50_) was defined as the concentration of inhibitor that decreased the larval mass by 50% compared to the control larvae.

#### 2.7.2. Larvicidal Activity

The larvae that were fed with BmPI (at different concentrations) were continuously maintained on fresh treated diet which was changed every 24 h. Larval mortality was recorded after 72 h of treatment. Four replicates were maintained for each treatment with 13 larvae per replicate (total, *n* = 52). Percent mortality was calculated using the formula of Abbott (1925) [21]. All the laboratory conditions were the same as in antifeedant activity study. Abbott's corrected mortality is calculated as [% mortality in treatment − % mortality in control/100 − % mortality in control] × 100. The lethal dose (LD_50_) was the concentration of BmPI that reduced the insect count to 50% of those fed on the control diet.

#### 2.7.3. Pupicidal Activity

The larvae which survived were continuously fed with normal diet until they became pupae and adults. Pupicidal activity was calculated by subtracting the number of emerging adults from the total number of pupae.

#### 2.7.4. Larval and Pupal Durations

The larvae which survived were continuously fed with normal diet. The larval duration was calculated after treated larvae became pupae. Pupal duration was calculated from the day of the emergence of adults from pupae.

## 3. BmPI Treatment and Estimation of Instar-Specific HGPs Activity

*H. armigera* exhibits differential gut proteinase activity at different larval instar stages; hence dose dependent inhibitory potential of BmPI was assessed against HGPs of larvae collected from infested fields of pigeon pea plants and reared on artificial diet containing BmPI.

## 4. Nutritional Parameters

Fourth instar larvae (*n* = 52, in four replicates for each treatment) were exposed to either 0.1% w/w BmPI-treated or a control diet for comparing a number of nutritional parameters. The larvae, feces, and leftover diet material were separated, dried, and weighted for calculating nutritional indices of consumption, digestion, and utilization of food [22, 23]. The nutritional indices, namely, efficiency of conversion of ingested food (ECI), efficiency of conversion of digested food (ECD), and approximate digestibility (AD), were calculated as follows: ECI = (Δ*B*/*I*) × 100; ECD = [Δ*B*/*I* − *F*] × 100; and AD = [(*I* − *F*)/*I*] × 100, where *I* = weight of food consumed; Δ*B* is change in body weight; *F* is weight of feces produced during the feeding period; metabolic cost (MC) was calculated as 100 − ECD.

## 5. Statistical Analyses

All extractions and analysis were carried out at least in four replicates and the means (with standard deviations) reported. Data collected were subjected to analysis of variance (ANOVA), and means of treatments were subjected to Fisher's least significant difference test.

## 6. Results and Discussion

### 6.1. Isolation, Purification, and Electrophoretic Analysis of BmPI

**Figure 1:**
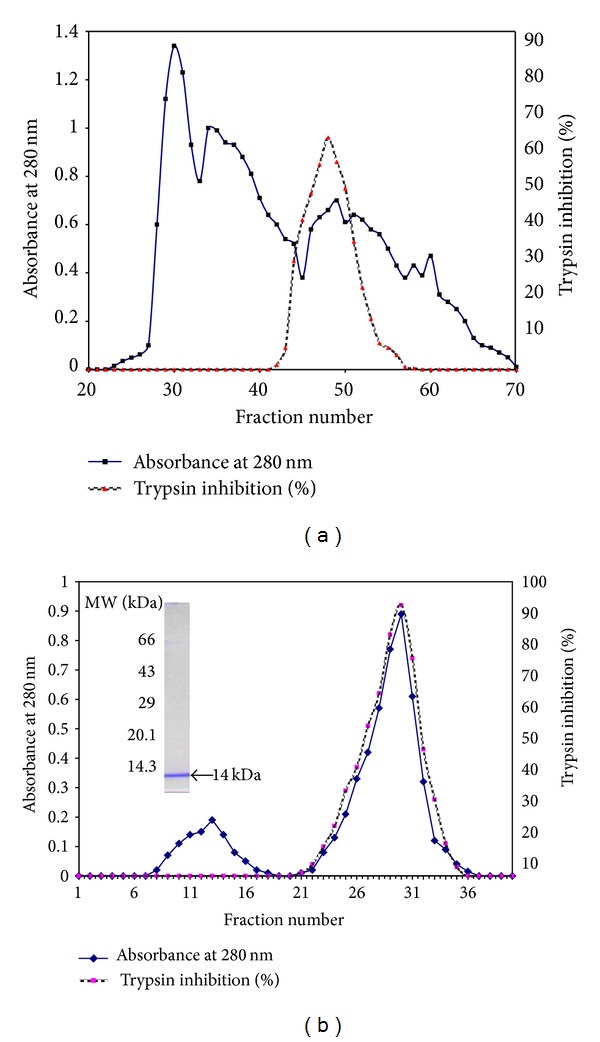
(a) Purification of BmPI using F_2_ dialyzed fraction on Sephadex G-75. Each fraction collected through the column is of 4.5 mL at an initial flow rate of 0.3 mL min^−1^. (b) Trypsin affinity purification of BmPI using fractions (42–58) from Sephadex G-75 and protein profile on 12% SDS-PAGE under reducing conditions. The retained BmPI was eluted with 100 mM HCl. Inset SDS-PAGE of pooled fraction (21 to 36): molecular weight markers are on the left and BmPI from trypsin affinity column chromatography are indicated on the right.

On comparison with other ammonium sulfate precipitated and dialyzed fractions (F_1_, F_3_), the fraction F_2_ exhibited strong inhibitory activity against trypsin and yielded peaks as shown in Figure 1(a) on Sephadex G-75 column. The active fractions (42–58) were pooled, dialyzed, lyophilized, and applied onto a Trypsin-Sepharose CL-4B column. Elution with HCl resulted in a major peak of BmPI with high inhibitory activity against bovine pancreatic trypsin (Figure 1(b)). The purification factor of the purified fraction was 9.1-fold more than that of crude extract with an yield of 7.68% (Table 1). The extracted protein was of ~14 kDa both in the presence and absence of dithiothreitol (Figure 1(b) inset).

### 6.2. *In Vitro *Effect(s) of BmPI on Midgut Proteinase(s) of* H. armigera*

**Figure 2:**
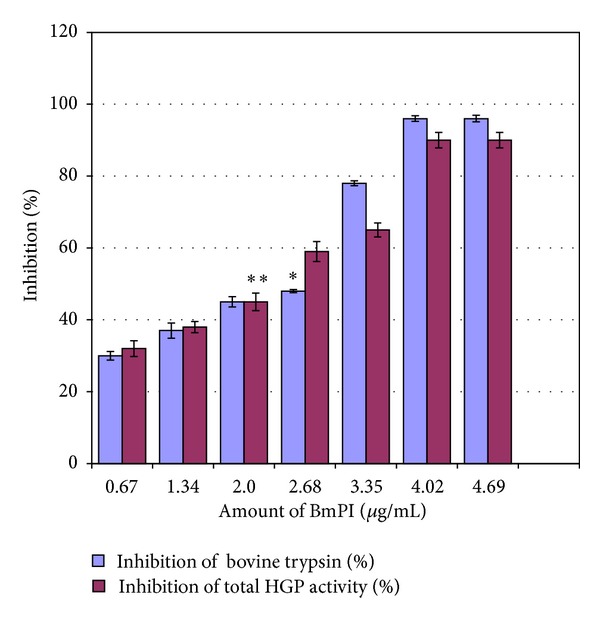
Inhibition of bovine trypsin and total proteolytic activity of HGPs from pigeon-pea fed larvae by BmPI: the assays were conducted using azocasein as substrate. The experiments were done in triplicates and mean was calculated. *IC_50_ value of BmPI against bovine trypsin was 2.68 µg/mL; **IC_50_ value against total HGPs was 2 µg/mL. IC_50_ is concentration of inhibitor, which reduces the enzyme activity to 50% of the original.

The digestive gut proteinases of* H. armigera* larvae during developmental growth are flexible, complex, and diverse. Therefore to assess the influence of BmPI on the midgut proteinases, fourth instar larvae were dissected and assayed with azocasein for the presence of proteinases. BmPI showed dose dependent inhibitory activity against total midgut proteolytic enzymes of* H. armigera*. However on comparison with inhibition profile of bovine trypsin with BmPI, the percent inhibition was (48%) for bovine trypsin and (59%) for total HGP at 2.68 µg concentration of BmPI. On further increasing the concentration of BmPI the bovine trypsin showed enhanced inhibition whereas the HGP activity decreased comparatively although not significantly (Figure 2). One of the possible reasons for this observation of higher activity towards general proteolysis may be the inhibitor inhibits proteinases* per se*, as well as those which possess activities similar to that of trypsin. To confirm that the inhibition of HGPs was due to the inhibitor protein (BmPI) and not because of a potential comigrating protein, the protein band corresponding to TI activity was electroeluted on preparative SDS-PAGE, dialyzed, and assessed for inhibition activity against insect gut proteinases and trypsin (data not shown).

As proteins are essential for completion of life cycle and it is exclusively during larval stages that intake and assimilation occur, it is advantageous for* H. armigera* larvae to maintain a constant level of digestive proteinases. Moreover, it is a physiological necessity to maintain the level of proteinase even in cases of poor protein diets [25]. Consequently, due to this higher inhibitory activity towards general proteolysis the effects on larval growth and physiology would be more pronounced.

In comparison to SKTI, BmPI was less effective whereas SBBI and AsPI showed comparable effectiveness against total midgut proteinases of* H. armigera* larvae (Table 2).

As inhibition reducing 90% activity of the gut enzymes of* H. armigera* larvae was achieved by BmPI, it was understandable that this inhibitor is a trypsin-like serine proteinase inhibitor which was confirmed by inhibition of bovine trypsin (Figure 2). Telang et al. (2003) reported inhibition of more than 80% of* H. armigera* proteinase activity with nonhost PIs from bitter gourd [26]. Moreover, PIs from tomato, which are highly stable to insect proteinases, inhibited about 50–80% proteinase activity of* H. armigera* larvae feeding on various host and nonhost plants. The IC_50_ of BmPI for* H. armigera* midgut proteinases was 2.0 µg/mL (Table 2) which is close to values reported for proteinase inhibitors from* Acacia senegal* (AsPI), soybean kunitz trypsin inhibitor (SKTI), and* Cicer arietinum* proteinase inhibitor (CaKPI) (<2 µg/mL) against trypsin enzyme of* H. armigera* [24] (however, as a cautionary note this comparison is only an approximation as HGP inhibition by BmPI is being compared with the inhibition of pure trypsin).

### 6.3. Inhibitory Activity of BmPI against Instar-Specific HGPs

**Figure 3:**
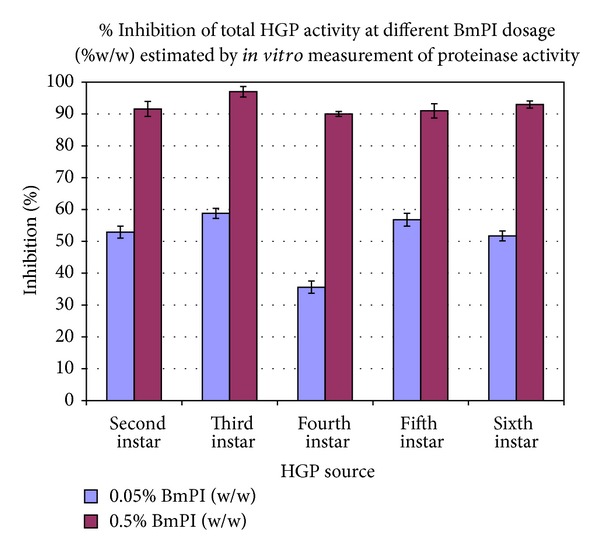
Prolonged treatment of larvae of second instar to sixth instar with two different concentrations of BmPI.

The percent inhibition of total HGP activity was variable among 2nd to 6th instar larvae. However, the 4th instar larvae were most insensitive to inhibition by BmPI at both concentrations (0.05 and 0.5% w/w) (Figure 3).

As compared to other instar stages, the fourth instar showed approximately 35.6% and 90% inhibition of total HGPs activity at 0.05% and 0.50% w/w dose of BmPI, respectively. This observation further supports the view that the 4th instar stage is the most voracious and devastating phase of* H. armigera* development. Moreover, it is in the 4th instar larval stage that maximum proteinase activity and maximum number of proteinase isoforms are expressed [27, 28].

### 6.4. *In Vitro* Stability of BmPI to HGPs

The stability of BmPI was measured against gut proteinases by incubating BmPI with HGPs for 45 min and 3 h [28]. The food retention time in the larval gut is 3 h and on treatment with BmPI for this duration, there was a slight decrease on the inhibitory potential of BmPI (data not shown). These results clearly demonstrate that BmPI is not only highly stable to insect gut proteinases but may also remain stable in the insect gut too: potato, bitter gourd, winged bean, peanut, and so forth. And other nonhost PIs also show similar results against* Helicoverpa* gut proteinases [26, 29]. The Kunitz and Bowman-Birk inhibitors lack alpha-helix, yet they vary greatly in their stability. Hydrophobic interactions of short stretches of hydrogen bonded sheets (soybean Kunitz trypsin inhibitor) stabilize Kunitz inhibitors [30], whereas in the Bowman-Birk inhibitors disulfide linkages minimize their conformational entropy and consequently enhance their stability [31].

### 6.5. Effect of BmPI on the Growth and Development of* H. armigera* Larvae

#### 6.5.1. Antifeedant, Larvicidal, and Pupicidal Activity

A strong antifeedant activity of 61% against* H. armigera* was observed at BmPI dosage of 0.5% w/w (Table 3). The larvae fed on BmPI supplemented diet showed a drastic reduction in mean larval weight to 178 mg/larvae as compared to control (356 mg/larvae) at dosage of 0.1% w/w reflecting the ED_50_ value. The weight loss was more pronounced at 0.1% dosage in comparison to 0.05% and 0.5% dosage of BmPI (Table 3).

The reduction in weight may be attributed to the nonutilization of amino acids essential for growth and development. Our findings are in conformity with the outcome of work reported by Telang et al. (2003) [26]. These effects were proved to be significant at *P* < 0.05. Further, these data are in conformity with the results of McManus and Burgess (1995) [32] where the mean weight of larvae fed on diet containing 0.2% or 0.5% (w/v) SBTI (soybean trypsin inhibitor) was significantly lower than the mean weight of larvae fed on standard chickpea diet.

The gut is the key site of digestive activities and serine proteinases are predominant in the gut of lepidopteran insects [33]. The inhibitory action of PIs suggests that they act to downregulate protein digestion significantly. Following a similar mode of action, the association of BmPI to* H. armigera* midgut may hinder the transport of enzymes and their hydrolyzed products and together in combination with a limited proteolysis may curtail the availability of amino acids consequently leading to poor larval growth, development, and high mortality.

**Figure 4:**
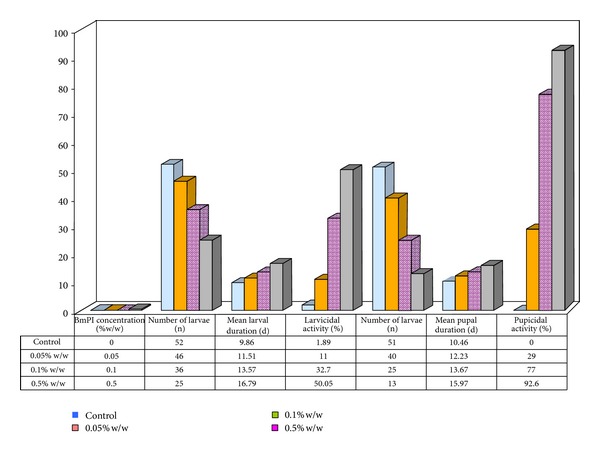
Effect of BmPI on* H. armigera* larval and pupal development. Number of larvae taken for study at each concentration (*n* = 52). d: days, n: number of larvae.

Antifeedants could be used to protect crops until slow acting biopesticides produce their effect [34]. The isolated proteinase inhibitor in this study clearly showed high antifeedant activity against* H. armigera* suggesting its greater susceptibility. Kuhar et al. (2013) had also reported antifeedant activity of a double headed peptidase inhibitor from* Dolichos biflorus *seeds [35]. In addition, BmPI (0.5% w/w) showed strong larvicidal and pupicidal activity of 50% and 92.6%, respectively (Figure 4).

Further, BmPI showed pronounced toxicity effect on the larvae of* H. armigera. *The larvae which were treated with lower dosage of BmPI showed higher amount of larval mortality. The LD_50_ value of BmPI was 0.5% w/w (Table 3).

#### 6.5.2. Larval and Pupal Durations and Assessment of Deformities and Fecundity

Larval developmental period was increased upon treatment with different concentrations of BmPI. In comparison to control (9.86 d) the BmPI treated* H. armigera* larvae exhibited an enhanced developmental duration of 11.51 d and 16.79 d when reared at concentration of 0.05% w/w and 0.50% w/w, respectively (Figure 4). Not only an increase in larval duration of* H. armigera* was observed but also there was an increase in pupal duration. The pupal duration of* H. armigera* development was 15.97 d studied at the maximum concentration of BmPI (0.5% w/w). This increase in the developmental duration over control group (10.46 d) is an indicator of the physiological changes taking place due to the presence of BmPI inhibitor in the feeding assay.

**Figure 5:**
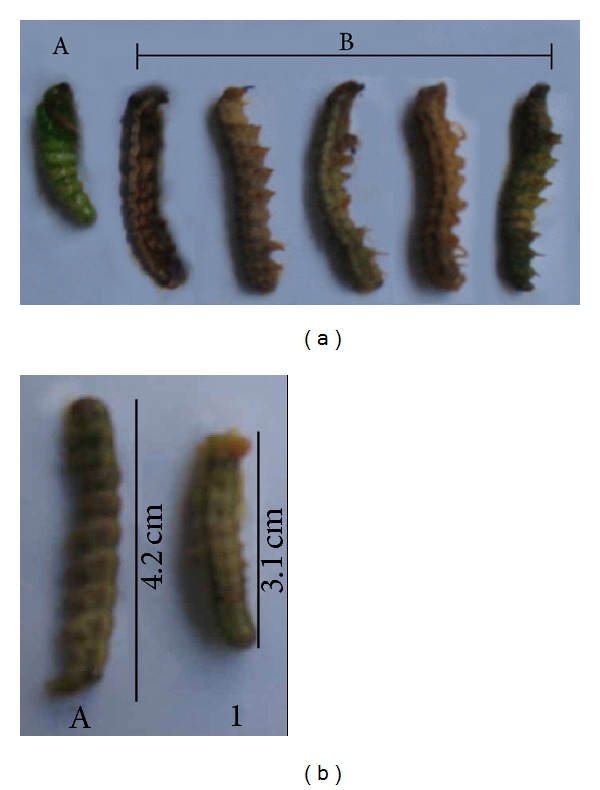
(a) Development of* H. armigera* fed on diets with and without BmPI. (A) Larvae fed on control diet show normal growth and pupation. (B) Larvae fed on test diet-containing BmPI did not undergo pupation and died. (b) Variations in the size (centimetres) of fourth instar* H. armigera* larvae fed on 0.1% w/w BmPI (1) and control (A) diets.

Larval and pupal durations were increased due to interference of proteinase inhibitor in the moulting process; consequently, the larvae were not able to go into further developmental stages of their life cycle. The presence of higher concentrations of BmPI in the diet reduced the larval size and caused several deleterious effects at all development stages of* H. armigera *larvae (Figures 5(a) and 5(b)).

The larvae were unable to continue normal physiological processes since the larvae consumed very low amount of diet. In general, prolonged larval-pupal durations were directly proportionate to the increase in pupicidal activities. This observation was corroborated with different degrees of abnormalities in the larval, pupal, and adult stages of* H. armigera* development. Around 92.5% malformed pupae (larval-pupal intermediate) and complete malformed adults (pupal-adult intermediate) were observed on the larvae fed on test diet 0.5% w/w of BmPI (Table 3). In addition the moulting was also delayed.

Larvae of* A. grandis* consuming SKTI exhibited similar growth and developmental patterns [36]. Such disturbances in the normal growth pattern may be attributed to the adverse effects of BmPI activity in inhibiting protein utilization and uptake which is important in metamorphosis [37]. The proteins localized in the insect cuticle are essential in the production of new adult tissues and enzymes [38]. Hopkins et al. (2000) [39] have suggested that these proteins are synthesized primarily from free amino acids following a reorganization of peptides from larval proteins without total degradation of amino acids. This reorganization necessarily involves proteolytic activity including that of serine and cysteine proteinases. Although, the actual* modus operandi *of these inhibitors is still debatable, it remains unclear that the manifestations of these deleterious effects of proteinase inhibitors is a fall out of antidigestive effect, through proteolysis inhibition [5], or a consequence of toxic effect by inducing over production of proteinases, leading to a crisis in the availability of amino acids [40]. Even the BmPI is casting similar challenge in the gut of* H. armigera* or possibly follows a different mode of action of inhibitors against insects which is responsible for larvicidal, pupicidal activities, deformity and retardation of metamorphosis.

An interesting observation was related to fecundity which adversely affected the moths emerging from BmPI-fed larvae. The downfall in egg laying capacity was dose dependent. The reduction observed was 63.78% and 95.91% at BmPI concentrations of 0.05% w/w and 0.5% w/w, respectively (Table 3). Moreover, BmPI also exhibited adverse effect on egg hatching capacities of* H. armigera* female moth. As compared to the control, significantly lower eggs were laid by female moths and the effect was dose dependent. At the maximum concentration of 0.5% w/w of BmPI the egg laying potential underwent dramatic reduction to 16 eggs (*N* = 2). Egg hatching too was remarkably reduced in BmPI-fed groups (Table 3). As compared to control (71%) egg hatching was dose dependent. At lower concentration of BmPI (0.05% w/w) the egg hatching was recorded to be 48% which declined to 13% at an increased concentration of 0.5% w/w. Thus, BmPI too was similar to PIs from other sources such as bitter gourd, winged bean, potato (PIN II), and groundnut which exhibited adverse effects on egg laying capacity and hatchability of* H. armigera *moth eggs [26, 31]. Gupta et al. (2002) [41] observed that fecundity of* H. armigera* was severely affected at 0.33% concentration of winged bean proteinase inhibitors in the artificial diet. The tomato PIs also cause dose dependent adverse effects on various developmental parameters of* H. armigera*, most notably on fecundity [27]. PIs extracted from nonhost source such as bitter gourd and experimented in artificial diet assays also manifest similar effect on* H. armigera* and* S. litura* [24]. Bitter gourd proteinase inhibitors (BGPIs) had detrimental effect on fertility and fecundity of* S. litura and H. armigera *larvae. The ingestion of BGPIs adversely affected the protein uptake at the larval stage, which was responsible for developmental abnormalities including reduction in the fertility and fecundity of the adults. During the different larval developmental stages significant accumulation of proteins is critical and crucial for vitellogenesis process. Thus, in accordance with the findings of other workers, BmPI may also be interfering in the process of vitellogenesis, thereby adversely affecting the growth and development of* H. armigera *larvae.

#### 6.5.3. Nutritional Parameters

*H. armigera* successfully completes its life cycle by adjusting and fine-tuning the metabolism process on nutritionally wide range of host plants. A high pupal mass is achieved by lepidopteran larvae supported on nutritive and nitrogen rich diets which is an essential factor in deciding the efficiency of pupation [42]. The concentration reflecting the values of ED_50_ (0.10% w/w BmPI) was used for analyses of dietary utilization. An increase in feeding was a consequence of the smaller larval size, since consumption was expressed as a ratio of body weight; the BmPI-fed larvae consumed 1.2× more than the control group (Table 4). Thus, BmPI apparently affected the consumption pattern. These results were followed by profound nutritional changes.

Nutritional analyses revealed that BmPI presented a toxic effect when ingested by larvae. An index of dietary utilization for* H. armigera* larvae showed reduced ECI and ECD and an increase in AD and MC on artificial diet supplemented with 0.10% w/w BmPI (Table 4). ECI and ECD decreased significantly by 43.23% and 53.88%, respectively, and AD and MC increased by 26.37% and 54.01%, respectively, as compared to* H. armigera* larvae reared on control diets. Further, on artificial diet incorporated with BmPI (0.10% w/w) food consumption by* H. armigera* larvae was enhanced by 20.58% as against the control. A drop in fecal production (~56.02% lower than that of the control) was observed on similar regimen of artificial diet. Table 4 shows that 0.10% w/w BmPI, incorporated into an artificial diet, decreases the growth, consumption rate, and fecal production of* H. armigera* larvae. These observations suggest that BmPI acts on intestinal tract and/or interferes with digestive capabilities of the insect or may be an outcome of physiological (postingestive) effects [43].

The reason behind a greater AD could be that an increase in demand for nutrients [44, 45] is compensated by the deficiency in foodstuff conversion (reduction in ECI and ECD). A downfall in ECI indicates that food is essentially being metabolized for generating energy and growth of insect is compromised due to limited conversion to body mass [44, 46]. As the proportion of digested food metabolized for energy increases, ECD decreases [47, 48]. Therefore, it is suggested that the reduction in ECD is a likely outcome by undermining the efficiency to convert foodstuffs into growth, perhaps by a diversion of energy from production of biomass into detoxification of BmPI, that is, an increase in costs as reported elsewhere [43, 49].

Plants generally produce PIs for countering insect attack, but insects like* H. armigera* are highly flexible in adapting to PI stress. This adaptation is an outcome of either overproduction of PI sensitive proteinases [50] and/or upregulating the expression of proteinases that are insensitive to the PIs produced by that plant [51] or inducing the production of PI-degrading enzymes. Dunse et al. (2010) [52] demonstrated that the combined inhibitory effect of NaPI (inhibitor from* Nicotiana alata*) and StPin1A (inhibitor from* Solanum tuberosum*) on* H. armigera* larval growth in the laboratory was reflected in the increased yield of cotton bolls in field trials of transgenic plants expressing both inhibitors. Therefore, it is suggestive that better crop protection can be achieved using combinations of inhibitors in which one class of PI is used to match the genetic capacity of an insect to adapt to a second class of PI and for such reasons exploring novel peptidase inhibitors will hold great promise in controlling* H. armigera* using PIs either independently or in combination(s).

## 7. Conclusions

*H. armigera* is one of the polyphagous insects and its larval stage is marked by active feeding for accumulating nutrients in completing its life cycle. As the insect enjoys feeding on diverse host plants survival chances are excellent. Any disturbance in protein metabolism especially due to the presence of PIs in the larval diet results in reduced fertility and fecundity. The ~14 kDa purified protein (BmPI) is a novel protein which performed a strong inhibitory activity against both the bovine pancreatic trypsin and HGPs.

Targeting the gut proteinases of* H. armigera* larvae with potent PIs from nonhost plants would be a wonderful approach in creating metabolic disturbances and consequently the growth and development of the insect itself. A serine PI from* B. monosperma* seeds has been identified to have significant potential in causing deleterious effects on the digestive proteinases, growth, and development of* H. armigera. In vitro* and* in vivo* studies have shown that BmPI is an effective, strong, and stable inhibitor that causes reduction in fecundity of* H. armigera*. Moreover, the BmPI adversely influences insect physiology which includes larval survival, weight, and both the efficiencies of conversion of ingested as well as digested food and approximate digestibility of* H. armigera* larvae, indicating that this protein has great toxic potential and therefore can be used as a peptidase inhibitor for controlling phytophagous insects such as* H. armigera*. However, we must consider the complexity of* H*.* armigera* gut proteinases and attention must be paid to select appropriate PIs genes with the gene-pyramiding strategy for inhibition of a diverse range of proteinases from the gut complex of lepidopteran pests.

## Figures and Tables

**Table 1:** Purification and percent recovery of BmPI from *B. monosperma* seeds.

Purification steps	Total volume (mL)	Total protein (mg)	Total trypsin inhibitory unit (TIU)	Specific activity (TIU/mg protein)	Purification factor	Yield (% recovery)
Crude inhibitor extract from seeds (100 gm)	300	2736.82	147744.79	53.98	1.00	100

Heat treatment (heat stable protein) at 60°C; 30 min	257	1935.23	144612.42	74.72	1.38	97.88

F_30–65%_ (NH_4_)_2_SO4 saturation fraction	15	512.34	41779.20	81.54	1.51	28.27

Gel filtration (Sephadex G-75) chromatography	2	68.26	16766.70	245.63	4.55	11.34

Trypsin-Sepharose CL-4B affinity chromatography	1	23	11298.98	491.26	9.1	7.68

^#^One inhibition unit is defined as the amount of the inhibitor required to inhibit 50% of trypsin activity, under the trypsin inhibition assay.

**Table 2:** IC_50_ (µg/mL) values for both PIs and standard PIs. Except for value of BmPI, others have been adapted from Babu and Subrahmanyam [24].

Enzyme source	Inhibitor	IC_50_ (µg/mL)*
*H. armigera* midgut proteinases	*B. monosperma* (BmPI)	2.0
	Standard SKTI (soybean Kunitz trypsin inhibitor)	0.13
	Standard SBBI (soybean Bowman-Birk inhibitor)	2.0
	*Acacia senegal* (AsPI)	2.0

*IC_50_: concentration of inhibitor, which reduces the enzyme activity to 50% of the original.

**Table 3:** Mean reduction in weight, percent survival, antifeedant activity, deformities, and fecundity at varying concentration of BmPI (% w/w). Calculated LD_50_ and ED_50_ values have been shown. Number of larvae taken for study at each concentration (*n* = 52). Each measurement was done in four replicates.

BmPI dosage (% w/w)	Weight (mg)	Survival^$^ (%)	Antifeedant activity (%)	Deformities (%)			Fecundity (*N* = 2)	
				Pupae	Adult insects	Eggs	Reduction in egg laying/female (%)	Egg hatching (%)
Control	356 ± 1.91	98 ± 2.12	0.0	4.5 ± 0.3	2.67 ± 0.6	392	0.0	71
0.05	297 ± 0.89	89 ± 1.33	0.0	31.4 ± 0.9	35.90 ± 0.9	142	63.78	48
0.10**	178 ± 2.31	67 ± 1.78	0.0	70.6 ± 0.6	68.90 ± 0.6	82	79.08	21
0.50*	98 ± 1.23	50 ± 0.89	61.15 ± 2.12	92.5 ± 0.6	100	16	95.91	13

* Indicates LD_50_ and **indicates ED_50_; *N*: two pairs of moths (two males and two females).

^$^Survival of larvae (%) = (number of insects alive in test/number of insects alive in control) × 100.

ED_50_: concentration of inhibitor that decreased the larval mass by 50% compared to the control larvae.

LD_50_: concentration of inhibitor that reduced the number of insects to 50% of those fed on control diet.

**Table 4:** Nutritional indices for *H. armigera* fourth-instar larvae fed on BmPI and control diet (*n* = 52).

Nutritional indices [relative change]	Treatment		Food consumption/larvae (mg dry weight basis)		Fecal production/larvae (mg dry weight basis)	
	Control	BmPI (0.10% w/w)	Control	BmPI (0.10% w/w)	Control	BmPI (0.10% w/w)
ECI (%) [43.23]	35.34 ± 0.50	20.06 ± 0.45	4.81	5.8	—	—
AD (%) [26.37]	70.67 ± 1.23	89.31 ± 1.12	—	—	—	—
ECD (%) [53.88]	50.03 ± 1.34	23.07 ± 1.14	—	—	1.41	0.62
MC (%) [54.01]	49.95 ± 0.97	76.93 ± 0.76	—	—	—	—

ECI: efficiency of conversion of ingested food; ECD: efficiency of conversion of digested food; AD: approximate digestibility; MC: metabolic cost; *n*: number of larvae taken for study at each concentration.

## References

[1] Heckel DG, Gahan LJ, Gould F, Daly JC, Trowell S (1997). Genetics of *Heliothis* and *Helicoverpa* resistance to chemical insecticide and to *Bacillus thuringiensis*. *Pesticide Science*.

[2] Liu Y, Sui Y-P, Wang J-X, Zhao X-F (2009). Characterization of the trypsin-like protease (Ha-TLP2) constitutively expressed in the integument of the cotton bollworm, *Helicoverpa* armigera. *Archives of Insect Biochemistry and Physiology*.

[3] Hilder VA, Gatehouse AMR, Sheerman SE, Barker RF, Boulter D (1987). A novel mechanism of insect resistance engineered into tobacco. *Nature*.

[4] Johnson R, Narvaez J, An G, Ryan C (1989). Expression of proteinase inhibitors I and II in transgenic tobacco plants: effects on natural defense against *Manduca sexta* larvae. *Proceedings of the National Academy of Sciences of the United States of America*.

[5] Jongsma MA, Bakker PL, Peters J, Bosch D, Stiekema WJ (1995). Adaptation of Spodoptera exigua larvae to plant proteinase inhibitors by induction of gut proteinase activity insensitive to inhibition. *Proceedings of the National Academy of Sciences of the United States of America*.

[6] Gatehouse AMR, Norton E, Davison GM, Babbé SM, Newell CA, Gatehouse JA (1999). Digestive proteolytic activity in larvae of tomato moth, *Lacanobia oleracea*; effects of plant protease inhibitors *in vitro* and *in vivo*. *Journal of Insect Physiology*.

[7] Jamal F, Pandey PK, Singh D, Khan MY (2013). Serine protease inhibitors in plants: nature’s arsenal crafted for insect predators. *Phytochemistry Reviews*.

[8] Pandey PK, Jamal F (2010). Identification and evaluation of trypsin inhibitor in the seed extracts of *Butea monosperma* (Flame of forest). *International Journal of Biotechnology and Biochemistry*.

[9] Laskowski M, Kato I (1980). Protein inhibitors of proteinases. *Annual Review of Biochemistry*.

[10] Richardson M, Rogers LJ (1991). Seed storage proteins: the enzyme inhibitors. *Methods in Plant Biochemistry, Amino Acids, Proteins and Nucleic Acids*.

[11] Jongsma MA, Beekwilder J (2011). Co-evolution of insect proteases and plant protease inhibitors. *Current Protein and Peptide Science*.

[12] Subramanian S, Mohankumar S (2006). Genetic variability of the bollworm, *Helicoverpa armigera*, occurring on different host plants. *Journal of Insect Science*.

[13] Tamhane VA, Chougule NP, Giri AP, Dixit AR, Sainani MN, Gupta VS (2005). *In vivo* and *in vitro* effect of Capsicum annum proteinase inhibitors on *Helicoverpa armigera* gut proteinases. *Biochimica et Biophysica Acta*.

[14] Erlanger BF, Kokowsky N, Cohen W (1961). The preparation and properties of two new chromogenic substrates of trypsin. *Archives of Biochemistry and Biophysics*.

[15] Lowry OH, Rosenbrough NJ, Farr AL, Randall RJ (1951). Protein measurement with the Folin phenol reagent. *The Journal of Biological Chemistry*.

[16] Laemmli UK (1970). Cleavage of structural proteins during the assembly of the head of bacteriophage T4. *Nature*.

[17] Brock FM, Forsberg CW, Buchanan-Smith JG (1982). Proteolytic activity of rumen microorganisms and effects of proteinase inhibitors. *Applied and Environmental Microbiology*.

[18] Lee MJ, Anstee JH (1995). Endoproteases from the midgut of larval *Spodoptera littoralis* include a chymotrypsin-like enzyme with an extended binding site. *Insect Biochemistry and Molecular Biology*.

[19] Giri AP, Kachole MS (1998). Amylase inhibitors of pigeonpea (*Cajanus cajan*) seeds. *Phytochemistry*.

[20] Ben Jannet H, Harzallah-Skhiri F, Mighri Z, Simmonds MSJ, Blaney WM (2000). Responses of *Spodoptera littoralis* larvae to Tunisian plant extracts and to neo-clerodane diterpenoids isolated from *Ajuga pseudoiva* leaves. *Fitoterapia*.

[21] Abbott WS (1925). A method of computing the effectiveness of an insecticide. *Journal of Economic Entomology*.

[22] Waldbauer GP (1968). The consumption and utilization of food by insects. *Advances in Insect Physiology*.

[23] Farrar RR, Barbour JD, Kenedy GG (1989). Quantifying food consumption and growth in insects. *Annals of the Entomological Society of America*.

[24] Babu SR, Subrahmanyam B (2010). Bio-potency of serine proteinase inhibitors from *Acacia senegal* seeds on digestive proteinases, larval growth and development of *Helicoverpa armigera* (Hübner). *Pesticide Biochemistry and Physiology*.

[25] Sarate PJ, Tamhane VA, Kotkar HM (2012). Developmental and digestive flexibilities in the midgut of a polyphagous pest, the cotton bollworm, *Helicoverpa armigera*. *Journal of Insect Science*.

[26] Telang M, Srinivasan A, Patankar A (2003). Bitter gourd proteinase inhibitors: potential growth inhibitors of *Helicoverpa armigera* and *Spodoptera litura*. *Phytochemistry*.

[27] Damle MS, Giri AP, Sainani MN, Gupta VS (2005). Higher accumulation of proteinase inhibitors in flowers than leaves and fruits as a possible basis for differential feeding preference of *Helicoverpa armigera* on tomato (*Lycopersicon esculentum* Mill, Cv. Dhanashree). *Phytochemistry*.

[28] Chougule NP, Hivrale VK, Chhabda PJ, Giri AP, Kachole MS (2003). Differential inhibition of *Helicoverpa armigera* gut proteinases by proteinase inhibitors of pigeonpea (*Cajanus cajan*) and its wild relatives. *Phytochemistry*.

[29] Harsulkar AM, Giri AP, Patankar AG (1999). Successive use of non-host plant proteinase inhibitors required for effective inhibition of gut proteinases and larval growth of *Helicoverpa armigera*. *Plant Physiology*.

[30] Bhattacharyya A, Babu CR (2009). Purification and biochemical characterization of a serine proteinase inhibitor from *Derris trifoliata* Lour. seeds: insight into structural and antimalarial features. *Phytochemistry*.

[31] Ramasarma PR, Appu Rao AG, Rao DR (1995). Role of disulfide linkages in structure and activity of proteinase inhibitor from horsegram (*Dolichos biflorus*). *Biochimica et Biophysica Acta*.

[32] McManus MT, Burgess EPJ (1995). Effects of the soybean (Kunitz) trypsin inhibitor on growth and digestive proteases of larvae of *Spodoptera litura*. *Journal of Insect Physiology*.

[33] Pauchet Y, Muck A, Svatoš A, Heckel DG, Preiss S (2008). Mapping the larval midgut lumen proteome of *Helicoverpa armigera*, a generalist herbivorous insect. *Journal of Proteome Research*.

[34] Murray KD, Alford AR, Groden E (1993). Interactive effects of an antifeedant used with *Bacillus thuringiensis* var San Diego delta-endotoxin on Colorado potato beetle (Coleoptera, Chrysomelidae). *Journal of Economic Entomology*.

[35] Kuhar K, Kansal R, Subrahmanyam B, Koundal KR, Miglani K, Gupta VK (2013). A Bowman-Birk protease inhibitor with antifeedant and antifungal activity from *Dolichos biflorus*. *Acta Physiologiae Plantarum*.

[36] Francoa OL, dos Santos RC, Batista JAN (2003). Effects of black-eyed pea trypsin/chymotrypsin inhibitor on proteolytic activity and on development of *Anthonomus grandis*. *Phytochemistry*.

[37] Chapman RF (1982). *The Insects, Structure and Function*.

[38] Missios S, Carter Davidson H, Linder D, Mortimer L, Okobi AO, Doctor JS (2000). Characterization of cuticular proteins in the red flour beetle, *Tribolium castaneum*. *Insect Biochemistry and Molecular Biology*.

[39] Hopkins TL, John Krchma L, Ahmad SA, Kramer KJ (2000). Pupal cuticle proteins of *Manduca sexta*: characterization and profiles during sclerotization. *Insect Biochemistry and Molecular Biology*.

[40] Broadway RM, Duffey SS (1986). Plant proteinase inhibitors: mechanism of action and effect on the growth and digestive physiology of larval *Heliothis zea* and *Spodoptera exiqua*. *Journal of Insect Physiology*.

[41] Gupta GP, Ranjekar PK, Birah A, Rani S, Gupta V, Sainani MN (2002). Effective inhibition of *Helicoverpa armigera* by non-host plant proteinase inhibitors. *ICAR News*.

[42] Awmack CS, Leather SR (2002). Host plant quality and fecundity in herbivorous insects. *Annual Review of Entomology*.

[43] Rodrigues Macedo ML, Durigan RA, da Silva DS, Marangoni S, Freire MDG-AM, Parra JRP (2010). *Adenanthera pavonina* trypsin inhibitor retard growth of *Anagasta kuehniella* (Lepidoptera: Pyralidae). *Archives of Insect Biochemistry and Physiology*.

[44] Koul O, Daniewski WM, Multani JS, Gumulka M, Singh G (2003). Antifeedant effects of the limonoids from *Entandrophragma candolei* (Meliaceae) on the Gram Pod Borer, *Helicoverpa armigera* (Lepidoptera: Noctuidae). *Journal of Agricultural and Food Chemistry*.

[45] Nathan SS, Kalaivani K, Murugan K, Chung PG (2005). Efficacy of neem limonoids on *Cnaphalocrocis medinalis* (Guenée) (Lepidoptera: Pyralidae) the rice leaffolder. *Crop Protection*.

[46] Singh D, Jamal F, Pandey PK (2014). Kinetic assessment and effect on developmental physiology of a trypsin inhibitor from *Eugenia jambolana* (jambul) seeds on *H. armigera* (Hübner). *Archives of Insect Biochemistry and Physiology*.

[47] Carlini CR, Grossi-de-Sa MF (2002). Plant toxic proteins with insecticidal properties. A review on their potentialities as bioinsecticides. *Toxicon*.

[48] Coelho MB, Marangoni S, Macedo MLR (2007). Insecticidal action of *Annona coriacea* lectin against the flour moth *Anagasta kuehniella* and the rice moth *Corcyra cephalonica* (Lepidoptera: Pyralidae). *Comparative Biochemistry and Physiology C: Toxicology and Pharmacology*.

[49] Wheeler DA, Isman MB (2001). Antifeedant and toxic activity of *Trichilia americana* extract against the larvae of *Spodoptera litura*. *Entomologia Experimentalis et Applicata*.

[50] de Leo F, Bonade-Bottino MA, Ceci LR, Gallerani R, Jouanin L (1998). Opposite effects on *Spodoptera littoralis* larvae of high expression level of a trypsin proteinase inhibitor in transgenic plants. *Plant Physiology*.

[51] Bolter C, Jongsma MA (1997). The adaptation of insects to plant protease inhibitors. *Journal of Insect Physiology*.

[52] Dunse KM, Kaas Q, Guarino RF, Barton PA, Craik DJ, Anderson MA (2010). Molecular basis for the resistance of an insect chymotrypsin to a potato type II proteinase inhibitor. *Proceedings of the National Academy of Sciences of the United States of America*.

